# A comparative study: classification vs. user-based collaborative filtering for clinical prediction

**DOI:** 10.1186/s12874-016-0261-9

**Published:** 2016-12-08

**Authors:** Fang Hao, Rachael Hageman Blair

**Affiliations:** University at Buffalo, 3435 Main Street, 706 Kimball Tower, Buffalo, 14214 USA

## Abstract

**Background:**

Recommender systems have shown tremendous value for the prediction of personalized item recommendations for individuals in a variety of settings (e.g., marketing, e-commerce, etc.). User-based collaborative filtering is a popular recommender system, which leverages an individuals’ prior satisfaction with items, as well as the satisfaction of individuals that are “similar”. Recently, there have been applications of collaborative filtering based recommender systems for clinical risk prediction. In these applications, individuals represent patients, and items represent clinical data, which includes an outcome.

**Methods:**

Application of recommender systems to a problem of this type requires the recasting a supervised learning problem as unsupervised. The rationale is that patients with similar clinical features carry a similar disease risk. As the “Big Data” era progresses, it is likely that approaches of this type will be reached for as biomedical data continues to grow in both size and complexity (e.g., electronic health records). In the present study, we set out to understand and assess the performance of recommender systems in a controlled yet realistic setting. User-based collaborative filtering recommender systems are compared to logistic regression and random forests with different types of imputation and varying amounts of missingness on four different publicly available medical data sets: National Health and Nutrition Examination Survey (NHANES, 2011-2012 on Obesity), Study to Understand Prognoses Preferences Outcomes and Risks of Treatment (SUPPORT), chronic kidney disease, and dermatology data. We also examined performance using simulated data with observations that are Missing At Random (MAR) or Missing Completely At Random (MCAR) under various degrees of missingness and levels of class imbalance in the response variable.

**Results:**

Our results demonstrate that user-based collaborative filtering is consistently inferior to logistic regression and random forests with different imputations on real and simulated data. The results warrant caution for the collaborative filtering for the purpose of clinical risk prediction when traditional classification is feasible and practical.

**Conclusions:**

CF may not be desirable in datasets where classification is an acceptable alternative. We describe some natural applications related to “Big Data” where CF would be preferred and conclude with some insights as to why caution may be warranted in this context.

**Electronic supplementary material:**

The online version of this article (doi:10.1186/s12874-016-0261-9) contains supplementary material, which is available to authorized users.

## Background

Recommender systems have been widely used to provide data driven suggestions for individuals [28]. The prediction of recommendations based on historical data from an individual, and data from individuals that are *similar* in their buying behaviors or preferences. Recommender system approaches can be broadly classified as either *content-based* or based on *collaborative filtering*. Briefly, content-based approaches infer a preferences structure of the individual based on detailed attributes of their personal preferences. In this setting, each item has an underlying attribute structure that can be leveraged for recommendations, e.g., Pandora uses hundreds of attributes to describe the essence of music [8]. In this work, we focus on the latter classification of recommender systems, *collaborative filtering*, which relies on the notion that individuals that agree on ratings of items are likely to also agree on ratings of other items, perhaps not known to them. Collaborative Filtering (CF) can be used to predict item ratings for an individual and to collectively develop a personalized *ranking* of items that may be of interest to them.

CF based recommender systems have enjoyed tremendous success in e-business, marketing, and for other personalized recommendation services [2]. Recently, recommender systems have emerged in the biomedical sciences. In these applications, the objectives are the same, predict ratings for missing items. However, in this case, items may represent clinical variables or diagnostic codes. Unfortunately, the translation from classic business applications to clinical utility is littered with basic challenges. Unlike marketing applications, clinical data is based on factors such as medical examination, clinical measurements, professional expertise, and may not necessarily be altered by the mindset or preferences of patients. This is an important distinction between the two application areas, as *user/patient* bias is less likely to play a role in medical applications. In marketing applications habitual high/low raters can skew prediction, and the data often requires adjustments or scaling. Another challenge is that the absence of a diagnosis (e.g., missing data) may indicate that a person has yet to be diagnosed, not necessarily that they do not have the disease. Recommender systems utilize a likert scale that is ordinal in nature, and this scale is uniform across all items. In contrast, clinical data can be a mixture of variable types (e.g., continuous, categorical, ordinal), which is more challenging to model and merge from different databases. The majority of applications of CF in the biomedical sciences have centered on the prediction of comorbidity from patient data consisting of diagnostic codes for diseases. The application to comorbidity prediction from diagnostic codes is quite natural given that recommender systems are often adopted to massive and sparse databases. There is also tremendous value in the standardization of these codes that enables seamless merging of databases. Davis et al. developed a Collaborative Assessment and Recommendation Engine (CARE) that relies on patient medical history as described by ICD-9-CM (International Classification of Diseases, Ninth Revision, Clinical Modification) codes for the prediction of future disease risk [13, 14]. CARE uses CF methods to predict each patients disease risk based on their own history, and the history of similar patients. The output is a patient-specific rank ordered list of diseases. CARE was applied to a subset of data from the medicare database consisting of 13 million patients with encouraging performance, which they believe could be improved by amending other features, such as, clinical and genetic data [9]. Folino et al. developed a similar approach to comorbidity, but add an additional layer with respect to prediction of disease risk that relies on association rules [18]. Recently, Folino et al. extended this approach and developed a COmorbidity-based Recommendation Engine (CORE), which extends their earlier model to include a clustering phase for patient records that aims to emphasize the local nature of the model [17]. CORE models also rely solely on ICD-9-CM codes.

Hassan et al. proposed an alternative application for CF in medical datasets [21, 32]. Their application in this area is fundamentally unique as it focusses on the use of CF for risk prediction using clinical data. The data consisted of a cohort of 4,557 patients from the MERLIN-TIME 36 trial [31] with acute coronary syndrome with measured features spanning clinical measurements, family history, and demographics. The overall objective was to predict outcomes such as sudden cardiac death and recurrent myocardial infraction. Unlike the comorbidity prediction described earlier, Hassan et al. consider an application with clear set of predictors, *X*, and an outcome, *Y*, which would traditionally be solved using classification methods. In their application, they utilize CF and compare the performance to logistic regression and support vector machines. The problem is therefore treated as an unsupervised learning problem, although traditionally a problem of this type would be cast as a supervised classification problem. Moreover, discretization is required in order to make use of CF, which ultimately leads to a loss of information.

Hassan et al. show that CF outperforms traditional classification methods on the MERLIN-TIME 36 trial data, which is not only promising for the use of CF with clinical data, but is a novel application of models that do not solely leverage diagnostic codes for diseases. However, untangling the advantages and disadvantages of CF in clinical applications of this type is challenging, not well understood, and likely very data dependent. We hypothesize that as the “Big Data” era progresses, there will be a natural draw to consider recommender systems, such as CF, and other scalable approaches, for biomedical data, which is growing in both size and complexity. This has motivated the present study, which takes steps to assess the performance of CF in different publicly available biomedical datasets. Our study, and Hassan et al., examine large, but not massive, datasets. However, there are natural implications for the use of CF in “Big Data” applications where classification is feasible and practical.

This paper assesses user-based CF recommender systems in the context of clinical risk prediction. Specifically, the problem of predicting the value for an unknown outcome and/or missing predictor variables is solved by leveraging patient similarity in a user-based CF algorithm. We compare recommender systems to logistic regression and random forests with different types of imputation. These algorithms are compared on four different publicly available data sets: National Health and Nutrition Examination Survey (NHANES, 2011-2012 on Obesity), Study to Understand Prognoses Preferences Outcomes and Risks of Treatment (SUPPORT), chronic kidney disease, and dermatology data. We have formulated a simulation pipeline that enables us to assess algorithm performance for these data across varying levels of missing data (low, moderate, severe). Our findings demonstrate CF based recommender systems are inferior for every dataset examined, and across each imposed level of missing data. Moreover, the difference between traditional classification machine learning approaches and CF is not marginal. This trend was also observed in simulated data with (and without) class imbalance in the response, with missingness that was Missing At Random (MAR) or Missing Completely at Random (MCAR). Our assessment is both consistent and sobering, and warrants the use of caution when considering user-based CF for the purpose of clinical risk prediction when traditional classification is an acceptable alternative.

## Methods

Recommender systems were compared to more traditional methods for imputation and classification on four different publicly available datasets with different levels of missing data. In this section, we briefly outline the data, algorithms, and how the assessment of performance was made.

### Data sets

Four different publicly available datasets were investigated, National Health and Nutrition Examination Survey (NHANES), Study to Understand Prognoses Preferences Outcomes and Risks of Treatment (SUPPORT), chronic kidney disease, and dermatology data. There are broad differences in these datasets that transcend beyond the scope of the study, and the population. These data vary in their size, both the number of predictors (*p*) and the number of observations (*N*), in some cases *N*>>*p*. Furthermore, there are missing data and heterogeneity in the population for many of the measured predictors. Each of the four data sets has different levels of *missingness* within predictor variables, ranging from less than 1% up to 16%. From this point of view, many of the features present in *Big Data* are present on a smaller scale in these data sets, but our simulations will make this more severe. Each dataset under investigation has a categorical outcome and can therefore be framed as a classification problem. Briefly, we detail each dataset below.

- ***National Health and Nutrition Examination Survey (NHANES, 2011-2012 on Obesity):*** The National Health and Nutrition Examination Surveys (NHANES) programs include several cross-sectional studies on the resident population of the United States related to nutrition and obesity [6]. NHANES includes a comprehensive set of dietary, social economic and biological information from participants and serves a wide range of public health objectives, including but not limited to disease prevalence and risk factors. We have focussed on the data in the 2011−2012 time period, which consists of 9,756 participants and 22 predictors (Additional file 1: Table S1). The current study focuses on the relationship between obesity and basic demographics, social economic status, smoking and drinking habits and physical activity. In our applications, the response variable is an indicator for obesity that is measured as a BMI of 30 or above. Participants that provided the responses *refuse to answer* or *do not know the answer* were eliminated from the dataset, rendering a total of 5,018 participants in the final analysis.
- ***SUPPORT Study:*** The SUPPORT (Study to Understand Prognoses Preferences Outcomes and Risks of Treatment) aims to estimate survival over a 180-day period and thus study the prognosis for hospitalized and seriously ill adults [11]. This prospective cohort study was carried out in 5 tertiary care academic centers in the United States. A total of 9105 patients were enrolled for Phase I and II trials. A total of 23 predictors (Additional file 1: Table S2) are used to build a predictive model, most of which are physiological measurements and physician evaluations of patient condition. The data were obtained from a collection provided by the Department of Biostatistics at Vanderbilt University [15].
- ***Chronic kidney disease:*** The data were collected in hospitals and can be used to predict chronic kidney disease through a set of 24 predictor variables, which includes age and 23 physiological measurements (Additional file 1: Table S3). There are 400 observations in the data set. The response variable is a binary indicator for chronic kidney disease. This data set is available through UCI machine learning repository (http://archive.ics.uci.edu/ml/) [1].
- ***Dermatology:*** The data were collected to classify eryhemato-squamous disease among six possible disease types. Such differential diagnosis has been a challenge in dermatology. There are 366 observations in the data and a set of 34 predictor variables, which include age, family history, clinical attributes and histopathological attributes (Additional file 1: Table S4). This data set is also available through UCI machine learning repository (http://archive.ics.uci.edu/ml/) [1].
- ***Simulated Data:*** In addition to the above real data sets, we also consider a simple simulation to mimic a controlled setting in which we investigate the performance of various methods for data that contains various degrees of class imbalance along with Missing At Random (MAR) or Missing Completely At Random (MCAR) data [29]. We performed two different sets of simulations, one to investigate performance under MAR and MCAR scenarios on a well balanced dataset (N=300), and another to investigate MAR and MCAR in larger datasets (N=1000) with class imbalance. The differences in sample size for the simulations was motivated by the desire to retain adequate support in the data under imbalanced settings with higher levels of missingness. Both simulations utilize a multivariate normal with *X*_*i*_∼*N*(0,1) for *i*=1,…5. The response was generated from this data using the least squares model *Y*=*X* *β*+*λ*·*ε*, where *β*=[1,1,0.1,0.1,0.1], *λ*=10^−^2, and *ε*∼*N*(0,1). The response was dichotomized at the mean. In our simulations of class imbalance, we consider simulations of severe, moderate, and low-moderate class imbalance, in which the minority class is represented at a rate of 20*%*, 25*%*, and 30*%*, respectively. The details of the MAR and MCAR mechanisms imposed on the data are provided in Simulation.

### Predictive model development

Each data set under consideration can be cast as a supervised learning problm. Our objective is to look comparatively at the use of recommender systems for the prediction of a clinical outcome against more traditional methods of classification and imputation. We focus the comparison on logistic regression [25] and random forests [4].

**Multiple Logistic Regression** is a statistical method for classification has a probabilistic interpretation for the assignment of classes [25]. Let *G*(*x*) be the predictor that partitions the model space into *K* distinct regions (or classes). Logistic regression models seek to estimate, *P*(*G*=*k*∣*X*=*x*), the posterior probability of a class assignment, *G*=*k*, given the data *X*=*x*. Following the formulation in [19], the logistic model is given as:

$$\begin{array}{@{}rcl@{}} Pr(G=k \mid X=x) &=& \frac{\exp(\beta_{k0}+{\beta_{k}^{T}}x)}{1+\sum_{l=1}^{K-1}\exp(\beta_{l0}+{\beta_{l}^{T}}x)},\\ &&k=1,\ldots,K-1,\\ Pr(G=K \mid X=x) &=& \frac{1}{1+\sum_{l=1}^{K-1}\exp(\beta_{l0}+{\beta_{l}^{T}}x)}. \end{array} $$

The parameters $\theta = \{\beta _{10}, {\beta _{1}^{T}}, \ldots, \beta _{(K-1)0}, \beta _{K-1}^{T}$} are usually fit using maximum likelihood approaches [25]. We evaluate the predictive accuracy via the misclassification rate, which is based on a 0−1 loss function. Datasets with a dichotomous response variable were fit using the glm function in the R programming language (https://www.r-project.org). The dermatology dataset has a multivariate response (six classes) and was fit using the glmnet package. The Hosmer-Lemeshow (HL) goodness of fit test [24] was used to assess goodness of fit for the dichotomous models. The HL test specifies the null hypothesis that the actual and predicted event rates are similar across quantiles of the data. Rejection of the null suggests that the actual and predicted rates are not the same and refinement of the model may be warranted. HL test was performed in the R programming language using the ResourceSelection package. In our applications, we used deciles and a threshold of *P*-value <0.05 to support a poor model fit. We also used a Brier score to assess calibration and goodness of fit in order to better compare with Random Forests, which is the mean squared difference between an individual and its predicted probability [33].

**Random Forest** is a machine learning technique that leverages ensemble learning for classification. that relies on aggregates over bootstrapped CART model [4]. CART models have been widely used for decision making in several research areas, e.g., medicine, engineering, and marketing [3]. Their popularity is due in part to their natural interpretation and flexibility.

**Fig. 1:**
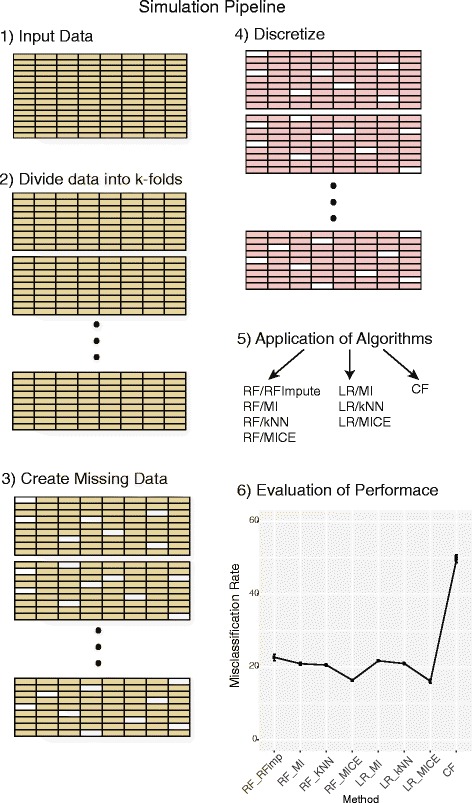
The simulation pipeline proceeds in a series of steps: (1) The data is input, and (2) divided into *K*-folds, (3) missing data is created at random, (4) the data is discretized, (5) a recommender system is fit along with variations of logistic regression and random forests, and the (6) performance is evaluated

Briefly, we motivate the random forest approach through the description of CART as a base classifier. The recursive partitioning algorithm examines each predictor variable in model, {*X*_1_,*X*_2_,…,*X*_*p*_}, for optimal split points that minimize loss subject to previous partitions in the model space. The process is depicted in Fig. 1 a for a simple two dimensional predictor space, {*X*_1_,*X*_2_}, and a two class outcome. Recursive partitioning can be viewed as a greedy-search for sub-regions in the model space that are good predictors of the outcome *Y*. The greediness arises from the fact that at each step, that splits are dependent on the splits that proceed them. For example, in Fig. 1 a, the first split divides the *X*_1_ region, and split 2 divides the *X*_2_ region, but the split is subject to the split that has already occurred, and so forth. Consequently, partitioning of this type can be visualized as a tree, where the splits are represented as internal nodes (Fig. 1 b). In our applications, we focus on classification tress, which bases prediction on the label of the majority class in the terminal nodes, or equivalently the sub-regions of the model space. The recursive partitioning is framed as an optimization problem that seeks to maximize the purity (of class) in the terminal regions [3]. The process of prediction is simply inputing an observation at the top of the tree and tracing it down to identify the appropriate terminal region and label. Unfortunately, CART models are known to be unstable, meaning that small changes in the training set can give rise to significantly different decision tree structures [19].

Random forests utilize CART models in an ensemble fashion to overcome instability and uncertainty in the population and predictor set. The *randomness* comes in two ways that relate to resampling of the training data and the predictor set. Briefly, each model in the ensemble is based on a sample that is bootstrapped with replacement from the training data. A decision tree is fit from the bootstrapped data. However, at each node in the tree, only a random subset of *m* predictors is considered for partitioning, instead of the entire set. Empirically, RFs have been shown to be relatively insensitive over different values of *m* [4]. In our applications, we have used $m=\sqrt {p}$, and have grown 5,000 trees for each implementation of the RF routine. Predictions for a RF are obtained by tracing the new sample down each decision tree in the ensemble and aggregating over those predictions. Unfortunately, due to the aggregate nature of the ensemble, the natural interpretation of the CART model is not retained. Calculations were performed in the R programming language using the randomForest package [4]. For dichotomous outcomes, the Brier score was calculated from the Out Of Bag (OOB) votes that arise from frequency prediction of classifications based on trees for which it was not in the bootstrap sample. Although this is not a probability, rather it is an OOB frequency of prediction, the Brier score is most often used for RF calibration [12].

**Collaborative filtering** is an algorithm that relies on user rating data for items to infer missing ratings for other users and items. This type of recommender system is widely used for the creation of *ranked lists* of items, that are *personalized* in the sense that the inference is based on other users with similar patterns of ratings. Applications to marketing are obvious. The scale of ratings is fixed across items. There are modifications to the standard CF that account for *user rating bias*, which occurs when individuals tend to always rate highly or poorly [28]. In certain applications this may be an issue, e.g., self-reporting, measurement, or doctor bias. The bias adjustment amounts to simply centering the rows (patients).

In the clinical application, users are patients, and items are derived from clinical features of the patients. Several of the measured features can be expected to be missing. We define the patients, *P*={*P*_1_,*P*_2_,…*P*_*n*_}, and the measured features on these patients *X*={*X*_1_,*X*_2_,…,*X*_*p*_}. This calculation is performed on the ratings over a common set of features, which have no missing values between the two patients. The similarity between patients *P*_*i*_ and *P*_*j*_ is defined as the cosine distance between their features:

1 $$\begin{array}{@{}rcl@{}} \text{sim}_{\text{cos}}(P_{i}(X), P_{j}(X)) &=& \frac{\langle P_{i}(X), P_{j}(X) \rangle}{||P_{i}(X)||||P_{j}(X)||}, \end{array} $$

where 〈·〉 denotes the inner product, and ||·|| is the euclidean norm.

**Fig. 2:**
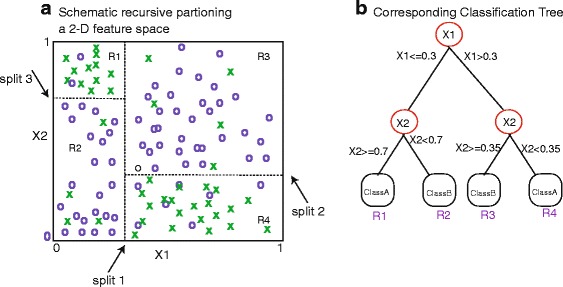
**a** A simplified example of a 2-D model space for variables *X*1 and *X*2. The recursive splitting of the model space identifies three splits in a sequential manner that optimally partition the data to minimize the prediction error for an outcome, *Y*. **b** The corresponding decision tree displays the split points (in A) as internal nodes in the tree. At each node, a binary question is asked, which in the continuous case, results in subsetting the variable range. The terminal nodes for a regression tree represent non-overlapping regions in the model space. The label given to a region is simply the majority class of the outcome, *Y*, in the region. Predictions for a *new sample* are obtained by tracing the new sample down the decision tree into a terminal region with a given label

Feature *X*_*i*_ of patient *P*_*j*_ is estimated as:

2 $$ \hat{X}^{P_{j}}_{i} =\frac{1}{\sum_{h\in N(P_{j})}\text{sim}_{\text{cos}}(P_{j}, h)} \sum_{h\in N(P_{j})} \text{sim}_{\text{cos}}(P_{j}, h)\cdot X_{h,i}  $$

where *h*∈*N*(*P*_*i*_) is the neighborhood centered on patient *P*_*i*_. A schematic depicting the notion of a neighborhood for a patient *P*_9_ is shown in Fig. 2 a. Missing data and outcome is predicted by aggregating across the *k* neighbors (Fig. 2 b). Note that CF does not treat the clinical prediction problem as supervised, rather it recasts a supervised learning one as an unsupervised problem. In the execution of CF on clinical data, the response variable *Y* is treated simply as another predictor, *X*_*i*_, with the prediction being made as a function of patient similarity (Eq. 2).

In our applications, the selection of the number of neighbors, *k*, was made based on 3-fold cross validation. Implementation of CF was performed using recommenderlab in the R programming language (https://www.r-project.org).

### Simulation

**Fig. 3:**
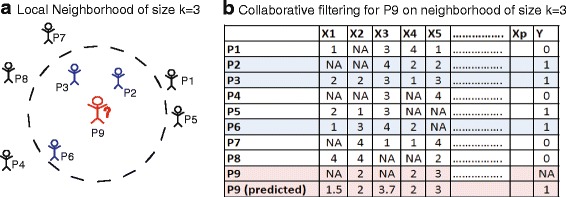
**a** A schematic of a local neighborhood for collaborative filtering of size *k*=3. The distance between individuals and patient 9 is quantified by a cosine distance. **b** The predicted recommendations for patient 9 are aggregate estimates over the neighborhood. In this simple example, the missing values are simply calculated as the average across the neighborhood, although in practice, the calculation is weighted by similarity

Our objective is to assess the performance of CF based recommender systems comparatively to logistic regression and random forests in clinical data with varying degrees of missingness. To this end, we have designed an experiment for manipulating the datasets to contain a percentage of missing values (NA). Each dataset was first examined at *baseline*, which is the data with *no additional missingness*. For the NHANES and dermatology data this corresponds to 3 and 5% missing at baseline, respecitively. Chronic Kidney and SUPPORT 10 and 16% were missing at baseline, respectively. On top of the baseline missingness, a percentage of the data was deleted at random to mimic low (<16%), moderate (20%), and severe (30%), levels of missingness. Our simulation proceeds in six phases (Fig. 3). (1) The data is input, and (2) divided into 3−folds, (3) missing data is created at random, (4) the data is discretized, (5) CF and classification models are fit, and the (6) performance is evaluated. Importantly, for each simulated setting steps 1–4 are performed, and the algorithms used for model fitting (step 5) utilizes same exact data in order to gain a fair assessment of their relative performance. We briefly detail each step in the simulation pipeline below.

1. ***Input data:*** The following datasets were input into the simulation pipeline: Chronic Kidney, Dermatology, NHANES, SUPPORT and simulated data.
2. ***Data divided into folds for cross-validation:*** Each dataset is divided into *K*=3 folds for cross-validation. The analyses were performed through 3-fold cross validation on each individual data set. Therefore, two thirds of the data were used as training data in each fold and the rest were used as test data. We utilize repeated cross-validation. In our applications to real data and simulation we repeat the cross validation process 50 times. For each run the folds are fixed throughout the simulation of different levels of missingness to achieve a cumulative affect.
3. ***Creation of missing data:*** For the real datasets, a fixed percentage of values in predictor variables were randomly deleted with the goal to simulate MCAR settings [22]. Since the deletion is random across all predictor variables, each is affected to a comparable extent. In all settings, the pattern of missing data is cumulative across the varying levels of severity. For example, for a given simulation, the missing values for the 20% simulation include, those missing in the 10% simulation. MAR was also imposed on the simulated data by creating a dependency between *X*_1_ on *X*_2_. Specifically, missingness was imposed on *X*_1_ if *X*_2_ was above a specified quantile. The specified quantile was adjusted as to let in varying levels of missingness. MCAR was also used in connection with the simulated data. The MCAR rate of missingness was matched to the MAR rate of missingness to enable fair comparisons. For each missing data scenerio, we simulated 50 unique patterns of missingness.
4. ***Discretization of continuous variables:*** Recommender systems are designed to utilize ratings, which are categorical or ordinal values by nature. Moreover, the number of levels for each variable in the predictor set is fixed and uniform over the set of predictors. The datasets under consideration contain a mixture of variable types. Notably, RFs can readily accommodate a mixture of variable types in the predictor set. However, in order to facilitate fair comparisons, the predictor variables that have continuous values were discretized. Specifically, for each of the four data sets, the maximal levels taken by categorical or ordinal variables were used to discretize continuous variables. For example, if data set *X* has 2 categorical variables that have values {1,2} and {1,2,3} respectively, the continuous variables in this data set will be discretized into three levels and take on values {1,2,3}. The threshold for discretization of a continuous variable is based on quantiles to ensure a *balance* between the discretization levels. Following these principles, NHANES data were subject to 7-level discretization, while SUPPORT and Chronic Kidney Disease data were subject to 5-level discretizations, and Dermatology data to 4-level discretization. Simulated data was also subject to 4-level discretization.
5. ***Application of algorithms for model fitting:*** CF and classification methods were applied to each data set as described above. Briefly, different types of imputation are described as follows: (1) *Mean imputation* was used for each predictor variable in the training data. Imputation values were calculated as the average of the non-missing values within this predictor variable. Subsequently, this mean value replaced all of the missing values for the corresponding variable. The mean value for the training data was also used for missing data in the test set. (2) *k-NN imputation* was implemented through preProcess and predict functions in the caret package in R. The missing predictor variable values in training data and testing data were filled in through k-NN imputation respectively. Cross validation (3-fold) was used for the selection of *k*. (3) *RF-Impute* is an imputation within the randomForest package in R. RF-impute begins with a median imputation, and the imputation is updated based on proximities after running an initial forest on the imputed data, see [5] for details. Missing values in testing data were imputed through mean imputation (as described above) instead of rf imputation. (4) Multivariate Imputation by Chained Equations (MICE) was applied using the R package mice [7]. MICE uses Gibbs sampling to complete a multivariate data set by iterating over a set of conditional densities representing the variabes in the dataset. Five datasets were imputed for each missing data setting.
6. ***Evaluation of Performance:*** Performance was based on the mean misclassification rate (0−1 loss) across all the folds, and the standard error of this mean estimate was calculated as standard deviation across the folds.

## Results

**Fig. 4:**
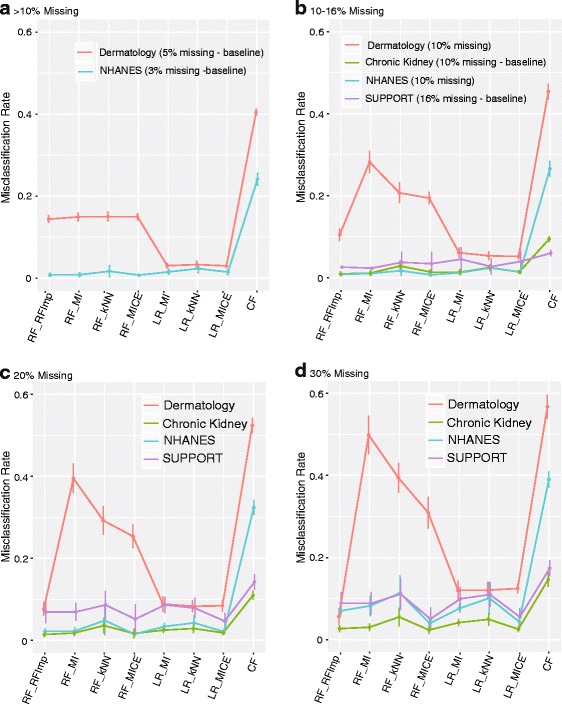
Simulation results for Chronic Kidney, Dermatology, NHANES, and SUPPORT are shown for **a** less than 10% missing data, **b** 10–16% missing data, **c** 20% missing data, **d** 30% missing data. Graphs depict the mean estimate of misclassification and standard error calculated via repeated 3-fold cross validation for 50 simulated patterns of missingness for each level of severity. The results for the baseline levels of missingness for each data sets is captured in **a** and **b**

Baseline and simulated scenarios of low, moderate, and severe missing data were implemented for the chronic kidney, dermatology, NHANES, and SUPPORT datasets. For each simulation the data was divided into 3-folds, missing data was created, discretization was performed. The following methods and imputations were evaluated: RF-RFImpute (RF Imp), RF with mean imputation (RF-MI), RF with kNN imputation (RF-kNN), RF with MICE imputation (RF-MICE), LR with mean imputation (LR-MI), LR with kNN imputation (LR-kNN), and LR with MICE (LR-MICE), and user-based CF. Performance was evaluated using the misclassification rate (Fig. 4), sensitivity (Table 1) and specificity (Table 2).

**Table 1:** Sensitivity for the most competitive classifiers and imputation combinations and CF

Dataset (% missing)	RF-MI	RF-MICE	LR-MI	LR-MICE	CF
NHANES (baseline=3%)	0.99	0.99	0.99	0.99	0.56
NHANES (10%)	0.98	0.99	0.98	0.99	0.63
SUPPORT (baseline = 16%)	0.99	0.98	0.98	0.97	0.98
Kidney (baseline =10%)	0.99	0.98	0.97	0.99	0.99
NHANES (20%)	0.98	0.98	0.99	0.99	0.49
SUPPORT (20%)	0.96	0.96	0.84	0.98	0.86
Kidney (20%)	0.98	0.99	0.98	0.98	0.98
NHANES (30%)	0.89	0.99	0.89	0.95	0.25
SUPPORT (30%)	0.86	0.99	0.81	0.93	0.82
Kidney (30%)	0.98	0.98	0.97	0.96	0.92

**Table 2:** Specificity for the most competitive classifiers and imputation combinations and CF

Dataset (% missing)	RF-MI	RF-MICE	LR-MI	LR-MICE	CF
NHANES (baseline=3%)	0.99	0.98	0.98	0.99	0.88
NHANES (10%)	0.97	0.96	0.99	0.99	0.88
SUPPORT (baseline = 16%)	0.98	0.98	0.99	0.98	0.91
Kidney (baseline = 10%)	0.99	0.99	0.94	0.99	0.85
NHANES (20%)	0.97	0.96	0.96	0.98	0.88
SUPPORT (20%)	0.97	0.96	0.97	0.97	0.85
Kidney (20%)	0.97	0.94	0.96	0.98	0.84
NHANES (30%)	0.93	0.93	0.94	0.96	0.83
SUPPORT (30%)	0.95	0.96	0.96	0.95	0.83
Kidney (30%)	0.98	0.98	0.95	0.97	0.82

For the real data, every variation of LR and RFs outperformed recommender systems for every data set in each scenario of missing data (Fig. 4). The dermatology dataset was by far the worst performing, which has six classes in the response variable. MI proves to be especially problematic for the dermatology data, especially when the the level of missingness increases (Fig. 4 c–d). For the large datasets (NHANES and SUPPORT) MI, kNN and MICE imputation led to only marginal differences in the misclassification rate when the level of missingness is low-moderate (Fig. 4 a–b). The advantages if MICE can be observed when the level of missingness is more severe (Fig. 4 b–c).

LR offers clear advantages over RF for the dermatology data. Otherwise, the performance of LR and RF is comparable, especially when used in connection with MICE (Fig. 4 c-d). The HL test indicates good calibration of LR models (*P*-value >0.05) (Additional file 1: Table S5). The HL test generally revealed improved calibration with increasing missingness. This was observed with NHANES for MI, kNN, and MICE, and with SUPPORT using MICE (Additional file 1: Table S5). This is not surprising, especially given the conditional nature of the imputation for MICE, but is potentially misleading as it may not reflect the underlying population well. The Brier score for RF and LR notably small, which also supports good calibration (Additional file 1: Table S6). The improving nature of the fit as a function of missingness was not observed in the Brier score as it was with the HL test.

For datasets with a dichotomous response, the sensitivity and specificity was calculated for CF, along with LR and RF for imputation methods, MI and MICE (Tables 1 and 2). Both the sensitivity and specificity of CF is often inferior for CF, with the exception of the sensitivity of the Kidney data with low-moderate levels of missingness (Tables 1 and 2). Generally, there is not a major difference or trend between in sensitivity or specificity that differentiates LR and RF for a given imputation method.

**Fig. 5:**
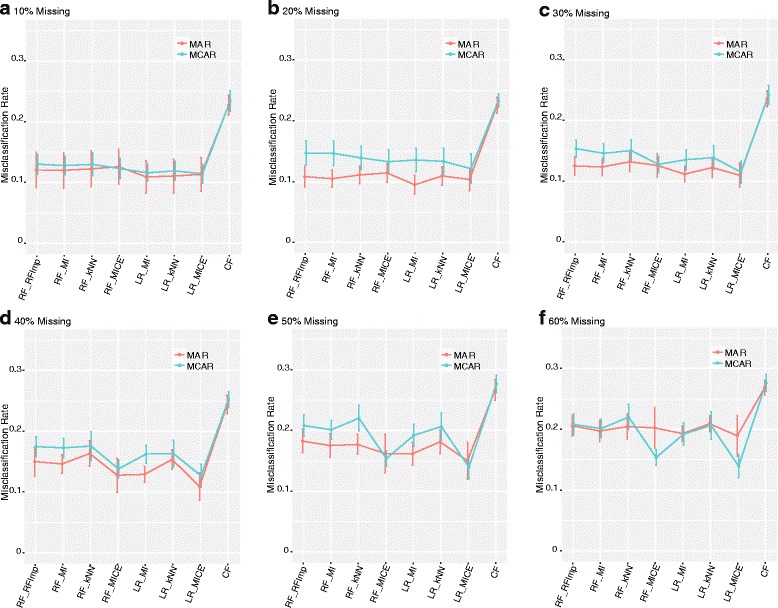
Results for simulated data under MAR and MCAR for **a** 10% missing data, **b** 20% missing data, **c** 30% missing, **d** 40% missing data, **e** 50% missing data and **f** 60% missing data. Graphs depict the mean estimate of misclassification and standard error calculated via repeated 3-fold cross validation for 50 simulated patterns of missingness for each level of severity

For the simulated datasets, we observed results that mimicked those of the real data for both MAR and MCAR. Specifically, CF was predominately poor when compared to traditional classification methods (Fig. 5). The MAR misclassification rate was slightly lower than the MCAR across increasing levels of missingness. The exception is that MAR performance degrades in the most severe missingness setting considered (Fig. 5 f). The MICE imputation was found to improve results considerably for MCAR in severe missingness settings when used in connection with RF or LR (Fig. 5 e, f). In more severe settings, MICE was also the superior imputation method for both MAR and MCAR (Fig. 5 d, e and f). MAR and MCAR patterns of missingness of the same type were also simulated on data with class imbalance. The overall results are consistent with the real data and balanced simulations. Traditional classification methods outperform CF in every setting (Additional file 1: Figure S1). The differences in performance between CF and traditional classification methods are much more pronounced in the severe imbalanced settings for both MAR and MCAR (Additional file 1: Figure S1G-I). Notably, for the traditional classification methods, the degree of imbalance had a lesser impact on performance compared to the level of missingness. In fact, with low levels of missingness (Additional file 1: Figure S1 A, D, G) MAR and MCAR are almost indistinguishable, although performance degrades slightly. Whereas, high class imbalance leads to more variability between methods (Additional file 1: Figure S1 C, F, I).

## Discussion

The objective of this study was to examine user-based CF on medical data with a categorical outcome. Hassan et al. evaluated CF on a rich dataset (<4,500 patients) for the prediction of adverse outcomes following a heart attack [21]. In this context, they demonstrated superior results (although slight) to competing statistical and machine learning methods for risk prediction. These comparisons were made to logistic regression models and support vector machines. The data inherently contained missing values, as was the case for the data in the present study, but additional missing data was not *pushed in* to the study and the focus was on a single dataset that is not publicly available. Our study has arrived at different conclusions regarding superiority of CF to classification methods. However, the overall study design and datasets are fundamentally different, and should be viewed as complimentary (not contradictory) to the work of Hassan et al.

At present, we are in the “Big Data” era, and it is becoming commonplace to reach for methods like CF, that are frequently used in other disciplines, to solve challenging problems in biomedical research. To this end, we anticipate more activity and attraction to this area. However, there remain many open questions regarding the utility of recommender systems on biomedical data for the purpose of clinical prediction, or more generally, classification. The study by Hassan et al. represents a novel framework for prediction of this type and has motivated further research in this direction [32, 34]. Their study, and ours, is limited in terms of size and uses cohorts from population studies or clinical trials. In our case, this was due to the lack of accessibility to medical databases, which are generally not publicly available.

The study by Hassan et al., and the present study, are fundamentally different to other research in the area that has centered on CF for comorbidity prediction on diseased codes [9, 17, 18]. It is natural to consider combining disease codes with additional attributes such as clinical data, patient history, and demographics to improve prediction. Data integration is a major challenge for “Big Data” and the translation of data to knowledge [26]. Although the present study is of small scale, we demonstrate the clinical data that can be modeled using traditional classification methods is preferable to CF. This study may be informative to developing and understanding approaches to data integration on a large-scale. On the other hand, there are several situations that may be inherent to a “Big Data” application that would prohibit the use of traditional classification methods. For example, the fusion of databases that have unified data representation, or the use of *real time* predictions that do not require re-training or tuning of the database, but rather merge new patient data in a seamless manner.

The present study was motivated by a desire to develop a more comprehensive understanding as to (1) how recommender systems perform on medical data, and (2) how this performance changes with an increased number of missing values. To address these questions, we set out to examine CF in a variety of controlled (simulate missing data) yet realistic (medical data sets) settings. We examined four different publicly available data sets, NHANES, SUPPORT, Chronic Kidney, and Dermatology. These datasets differed in both scope and size, but each had an outcome and could be framed as a classification problem.

Our simulation pipeline involved division into folds, creation of missing data, discretization, application of classifiers, and the evaluation of performance. Our simulation approach has exposed some major weaknesses in CF based recommender systems for prediction in medical data, but is not without limitations. Notably, there is not a *one size fits all* solution to classification problems. We selected logistic regression and random forests, but there are several other classification methods that could be used in this context. Importantly, it was not our objective to compare performance between classifiers, but rather to evaluate traditional and cutting edge classification methods as an appropriate alternative to recommender systems. Logistic regression is perhaps the most widely used statistical model for classification with a dichotomous response variable. Random Forests are a machine learning approach that leverages ensemble learning of CART models for classification. Recently, in a comprehensive study RF was found to be superior overall when compared to 179 different classifier on 121 datasets in the UCI machine learning repository [16]. RF is an attractive competitor for this study because of the ensemble nature, ability to handle missing data, and it is generally robust to noise and outliers [4]. Moreover, variations of the RF approach have been shown to be effective in “Big Data” settings, such as electronic health records [27]. Had recommender systems not been consistently inferior, a deeper investigation of alternative classification methods would have been warranted.

Another limitation of our study is the amount and pattern of missing data. Limitations on the amount of missing data were largely a function of the CF implementation in recommenderlab, where 30% was the maximum that could be achieved without errors related to the identification of *k* nearest neighbors (even for small *k*). The *missingness* of the data was simulated as missing completely at random. In realistic settings, this may not be the case, especially in databases housing electronic health records. However, the simulation of *not missing at random* is notably more difficult and subject to intense bias. Finally, a discretization of the data is required for recommender systems. Our approach to discretization was to have it dictated by the number of levels in non-continuous variables, and assigning the data according to quantiles. However, recommender systems often work on a likert scale and are ordinal in nature. We found this makes the discretization process rather awkward for medical data. In general, the discretization process results in a substantial amount of information loss. To this end, LR models and RFs are inherently flexible in that mixed predictors (continuous, categorical, etc.) can be accommodated. However, in order to level the playing field and facilitate the most fair comparisons, we discretized (unnecessarily) the continuous predictors. Application of classifiers to the original (non-discretized) data would have led to improvements in performance, and consequently widened the gap between CF and traditional classification methods. The relative size of the classes for the response variable also influences performance, especially in situations of severe imbalance. Our simulations showed that CF was particularly sensitive to class imbalance (Additional file 1: Figure S1). One possibility for this is the discretization will be negatively impacted. In our simulated data, we also discretized continuous variable for traditional classification methods. However, for CF, we hypothesize that the discretization has more of an impact on the model due to the nature of the the class assignment and the dependency on similarities (Eq. 1–2). The clinical data that we considered was relatively well balanced, with most severe imbalance for NHANES (35% minority class rate). Since imposing class imbalance on the real data would require subsetting or resampling the data, and consequently cutting down the sample size, we examined the impact of class imbalance on simulated data with missingness that is MAR or MCAR. Therefore, CF did not appear to offer any obvious advantage in these settings, but teasing out the contributions of the imbalance and the missingness in a real clinical data set would prove to be more challenging, and will be an area of future research. Notably, RFs have shown promise in class imbalance problems via down sampling and weighted loss [10], and we hypothesize that they would be generally more effective in imbalanced settings.

The size of the feature space is a major consideration. If the feature space is high-dimensional (*N*<<*p*) there are many conceptual issues that arise with the concept of nearest neighbor that are rooted in the inherent sparsity of the feature space [19, 23]. The proximity of a neighbor increases considerably as the size of feature space increases. The *local* nature of k-NN calls into question the value and quality of a neighbor [19]. In the context of a rich marketing database, issues related to the dimension of the feature space issue are often secondary to the extreme sparsity of the data. However, in the case of medical data, the issue of poor neighbor quality may not only arise, but may also be masked by the discretization process. This would certainly be the case in the classic “Big Data” settings, where the population itself is severely heterogenous. These weaknesses for large, sparse databases are also recognized in more classical, non-medical applications [20, 30].

Lack of stability and quality of the neighbor is also reflected in the implementation of RFs with kNN imputation with severe missing data for dermatology (Fig. 4 d). On the other hand, CF did not exhibit this instability, although the performance was uniformly poor. The underlying models for kNN imputation in a RF and CF based recommender systems are essentially identical in how the predictor set is imputed. The difference lies in how the response is handled. The problem is treated as a supervised one for kNN imputation in a RF, and unsupervised for CF based recommender systems. Generally, re-casting problems that are unsupervised as supervised is a popular *trick* in data mining, as there are several advantages due to the fact that there is an outcome, and *loss* can be measured [19]. On the contrary, casting a problem that is supervised as unsupervised, as in this approach, does not offer the same advantages.

Our focus is restricted to user-based CF under basic assumption that the number of levels for the variables are equivalent. The cosine distance is often used for a similarity measure. Pearson correlation is another popular choice. In our applications, there were negligible differences between the two. Another consideration is that alternative similarity measures can be used that would enable more flexibility in terms of the variable constraints. Likewise, variations of CF may lead to improved performance and is a topic of future research [2]. Regardless, the *quality of neighbor* issue would still remain for alternative similarities and methods. Therefore, we hypothesize that datasets modeled using traditional classification methods will likely achieve better performance when compared to CF-based methods.

## Conclusions

In summary, our results consistently put CF in a poor light for clinical prediction. We observed overwhelming evidence that traditional classification methods outperform user based-CF in simulation and real clinical datasets, across different levels of missing data mechanisms of missingness, as well as class imbalance in the response variable. The results of this work call into question CF as a general strategy for risk prediction in datasets where classification is an acceptable alternative [21, 32, 34]. In this setting, recasting a supervised learning problem as unsupervised was demonstrated to be suboptimal. This is not to say that CF does not, and will not, have utility for medical data. Scalability, dynamic learning, and merging of database are practical challenges that make CF an attractive option. However, we strongly suggest exercising caution if the objective is classification, and the size of the data can be accommodated with traditional classification methods or alternative machine learning approaches.

## Additional file

Additional file 1 Contains Supplemental Figure 1 and Supplemental Tables 1–6.

## Abbreviations

CART: Classification and regression tree
kNN: k-nearest neighbors
LR: Logistic regression
LR- MI: Logistic regression with MICE imputation
LR- MICE: Logistic regression with MICE imputation
MAR: Missing at random
MCAR: Missing completely at random
MI: Mean imputation
MICE: Multivariate imputation by Chained equations
MNAR: Missing not at random
NHANES: National health and nutrition examination survey
RecSys: Recommender systems
RF: Random forest
RF-kNN: Random forest with k-nearest neighbor imputation
RF-MI: Random forest with mean imputation
RF- MICE: Random forest with MICE imputation
RF- RFImp: Random forest with random forest imputation
SUPPORT: Study to understand prognoses preferences outcomes and risks of treatment
