# Supplementary material for: A comparative study: classification vs. user-based collaborative filtering for clinical prediction
Source: BMC Med Res Methodol. 2016 Dec 8;16:172. doi: 10.1186/s12874-016-0261-9 (PMC5146891; doi:10.1186/s12874-016-0261-9)
Supplement: Additional file 1 — Contains Supplemental Figure 1 and Supplemental Tables 1–6. [file 12874_2016_261_MOESM1_ESM.pdf]

Supplemental Figures

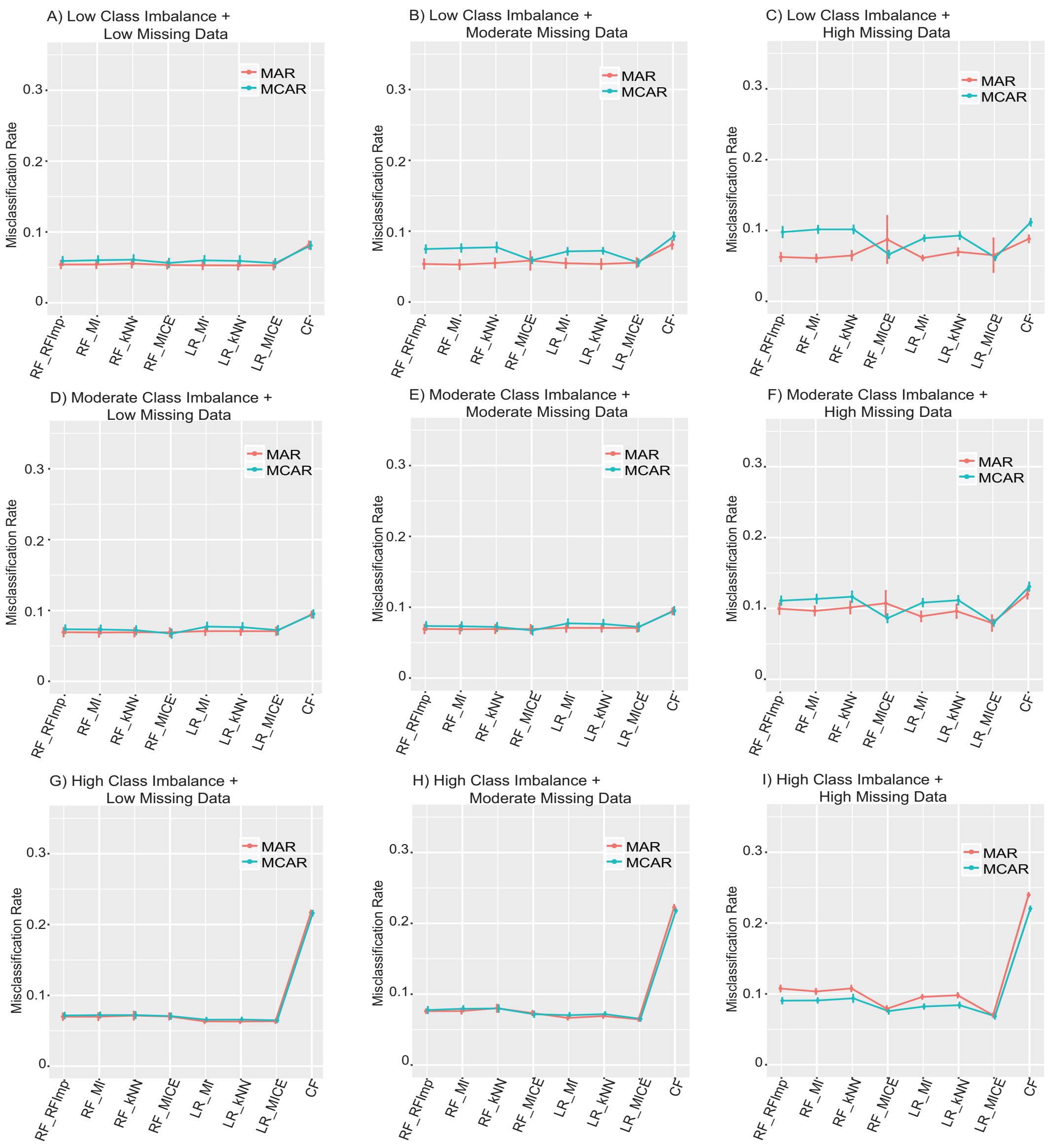

Figure 1: Results for simulated class low (A-C), moderate (D-F) and high class imbalances (G-I) , which were achieved by setting the minority class rate at 30%, 25%, and 20%, respectively. Missing data imposed via MAR and MCAR mechanisms were examined in these settings, the levels of severity are given as low (10% missing), moderate (30% missing) and high (60% missing). These different levels of missingness (columns) are shown for each class imbalance setting (rows).

Supplemental Tables

Variable	Description
RIAGENDR	gender
RIDAGEYR	Age at screening
RIDRETH3	Race
DMQMILIZ	Served active duty in US Armed Forces
DMDBORN4	Country of birth (US, other countries)
DMDEDUC2	Education level for adults 20 yo or older
DMDMARTL	Marital Status
INDHHIN2	Annual household income
ALQ101	Had at least 12 alcohol drinks/1 yr?
CBD070	Money spent at supermarket/grocery store
CBD090	Money spent on nonfood items
CBD110	Money spent on food at other stores
CBD120	Money spent on eating out
CBD130	Money spent on carryout/delivered foods
PAD680	Minutes sedentary activity
PAQ710	Hours watch TV or videos past 30 days
PAQ715	Hours use computer past 30 days
SMQ020	Smoked at least 100 cigarettes in life
SMQ680	Used tobacco/nicotine last 5 days?
WHQ030	How do you consider your weight
WHQ040	Like to weigh more, less or same
WHD140	Self-reported greatest weight (pounds)
Obese	Response variable: obese or not, i.e. whether BMI being 30 or above

Table 1: Description of Variables included in analysis for NHANES

Variable	Description
erythema	Erythema (0, 1, 2 or 3)
scaling	Scaling (0, 1, 2 or 3)
definite_borders	Definite borders (0, 1, 2 or 3)
itching	Itching (0, 1, 2 or 3)
koebner_phenomenon	Koebner phenomenon (0, 1, 2 or 3)
polygonal_papules	Polygonal papules (0, 1, 2 or 3)
follicular_papules	Follicular papules (0, 1, 2 or 3)
oral_mucosal_involvement	Oral mucosal involvement (0, 1, 2 or 3)
knee_and_elbow_involvement	Knee and elbow involvement (0, 1, 2 or 3)
scalp_involvement	Scalp involvement (0, 1, 2 or 3)
family_history	Family history (0 or 1)
melanin_incontinence	Melanin incontinence (0, 1, 2 or 3)
eosinophils_in_infiltrate	Eosinophils in the infiltrate (0, 1, 2 or 3)
PNL_infiltrate	PNL infiltrate (0, 1, 2 or 3)
fibrosis_of_papillary_dermis	Fibrosis of the papillary dermis (0, 1, 2 or 3)
exocytosis	Exocytosis (0, 1, 2 or 3)
acanthosis	Acanthosis (0, 1, 2 or 3)
hyperkeratosis	Hyperkeratosis (0, 1, 2 or 3)
parakeratosis	Parakeratosis (0, 1, 2 or 3)
clubbing_of_rete_ridges	Clubbing of the rete ridges (0, 1, 2 or 3)
elongation_of_rete_ridges	Elongation of the rete ridges (0, 1, 2 or 3)
thinning_of_suprapapillary_epidermis	thinning of suprapapillary epidermis (0, 1, 2 or 3)
spongiform_pustule	Spongiform pustule (0, 1, 2 or 3)
munro_microabcess	Munro microabcess (0, 1, 2 or 3)
focal_hypergranulosis	Focal hypergranulosis (0, 1, 2 or 3)
disappearance_of_granular_layer	Disappearance of the granular layer (0, 1, 2 or 3)
vacuolisation_and_damage_of_basal_layer	Vacuolisation, damage of basal layer (0, 1, 2 or 3)
spongiosis	Spongiosis (0, 1, 2 or 3)
saw_tooth_appearance_of_retes	Saw-tooth appearance of retes (0, 1, 2 or 3)
follicular_horn_plug	Follicular horn plug (0, 1, 2 or 3)
perifollicular_parakeratosis	Ferifollicular parakeratosis (0, 1, 2 or 3)
inflammatory_monoluclear_inflitrate	Inflammatory monoluclear inflitrate (0, 1, 2 or 3)
band_like_infiltrate	Band-like infiltrate (0, 1, 2 or 3)
age	Age (continuous)
class	Class (six different disease classes)

Table 2: Description of Variables included in analysis for UCI Dermatology Data

Variable	Description
age	Age(numerical) in years
bp	Blood Pressure(numerical) in mm/Hg
sg	Specific Gravity(nominal) sg in (1.005,1.010,1.015,1.020,1.025)
al	Albumin(nominal) (0,1,2,3,4,5)
su	Sugar(nominal) (0,1,2,3,4,5)
rbc	Red Blood Cells(nominal) (normal,abnormal)
pc	Pus Cell (nominal) (normal,abnormal)
pcc	Pus Cell clumps(nominal) (present,notpresent)
ba	Bacteria(nominal) (present,notpresent)
bgr	Blood Glucose Random(numerical) in mgs/dl
bu	Blood Urea(numerical) in mgs/dl
sc	Serum Creatinine(numerical) in mgs/dl
sod	Sodium(numerical) in mEq/L
pot	Potassium(numerical) in mEq/L
hemo	Hemoglobin(numerical) in gms
pcv	Packed Cell Volume(numerical)
wbcc	White Blood Cell Count(numerical) in cells/cumm
rbcc	Red Blood Cell Count(numerical) in millions/cmm
htn	Hypertension(nominal) (yes,no)
dm	Diabetes Mellitus(nominal) (yes,no)
cad	Coronary Artery Disease(nominal) (yes,no)
appet	Appetite(nominal) (good,poor)
pe	Pedal Edema(nominal) (yes,no)
ane	Anemia(nominal) (yes,no)
class	Class (nominal) (chronic kidney disease, no disease)

Table 3: Description of Variables included in analysis for UCI Chronic Kidney Disease Data

Variable	Description
scoma	SUPPORT Coma Score based on Glasgow D3
sps	support physiology score day 3
aps	APS III no coma, imp bun,uout for ph1,D3
surv6m	6M model survival prediction at day 3
hday	Day in Hospital at Study Admit
prg6m	MD 6 Month Survival Estimate
meanbp	Mean Arterial Blood Pressure Day 3
wblc	White Blood Cell Count Day 3
hrt	Heart Rate Day 3
resp	Respiration Rate Day 3
temp	Temperature (celcius) Day 3
pafi	PaO2/(.01*FiO2) Day 3
alb	Serum Albumin Day 3
bili	Bilirubin Day 3
crea	Serum creatinine Day 3
sod	Serum sodium Day 3
ph	Serum pH (arterial) Day 3
glucose	Glucose Day 3
bun	BUN Day 3
urine	Urine Output Day 3
dtime_label	Whether survived more than 180 days or not

Table 4: Description of Variables included in analysis for SUPPORT Data

Data	missing %	LR-MI	LR-KNN	LR-MICE
NHANES	base	0.273	0.226	0.231
NHANES	10%	0.371	0.242	0.349
NHANES	20%	0.328	0.213	0.310
NHANES	30%	0.361	0.211	0.361
SUPPORT	N/A			
SUPPORT	16%	0.612	0.659	0.743
SUPPORT	20%	0.622	0.671	0.762
SUPPORT	30%	0.690	0.603	0.711
Chronic Kidney	N/A			
Chronic Kidney	10%	0.935	0.967	0.966
Chronic Kidney	20%	0.962	0.981	0.935
Chronic Kidney	30%	0.994	0.985	0.965

Table 5: P-values from the Hosmer-Lemeshow goodness of fit test. P-value< 0.05 indicates evidence of a poor fit.

	RF RF-Imp	RF-MI	RF-kNNImp	RF-MICE	LR-MI	LR-kNNImp	LR-MICE
NHANES (base)	0.01	0.01	0.02	0.01	0.02	0.02	0.02
NHANES (10% NA)	0.01	0.01	0.02	0.01	0.01	0.02	0.01
NHANES (20% NA)	0.02	0.02	0.05	0.02	0.03	0.04	0.02
NHANES (30% NA)	0.07	0.08	0.11	0.04	0.08	0.10	0.04
SUPPORT (base)	0.03	0.02	0.04	0.03	0.05	0.03	0.04
SUPPORT (10%)	0.07	0.07	0.09	0.04	0.09	0.08	0.05
SUPPORT (20%)	0.09	0.09	0.11	0.03	0.10	0.11	0.05
Kidney (base)	0.01	0.01	0.03	0.01	0.01	0.02	0.01
Kidney (20%)	0.01	0.02	0.04	0.02	0.03	0.03	0.02
Kidney (30%)	0.03	0.03	0.05	0.02	0.04	0.05	0.03

Table 6: Brier score for RF and LR models. Smaller score indicates superior goodness of fit.
